# Effects of Probiotic and Dietary Fiber Supplementation on Metabolic Syndrome-Related Features, Mood, and Sleep in Adults with Obesity

**DOI:** 10.3390/nu18121851

**Published:** 2026-06-09

**Authors:** Jieping Wang, Fuyi Ma, Pei-Fang Wang, Chi-Hsiang Hung, Kun-Tien Wu, Li-An Liao, Jia-Yu Guo, Chien-Wen Hou

**Affiliations:** 1 School of Exercise and Health, Shanghai University of Sport, Shanghai 200438, China; wangjieping94@126.com (J.W.); 18505093326@163.com (F.M.); 2 Laboratory of Exercise Biochemistry, Department of Sports Sciences, University of Taipei, Taipei 11153, Taiwan; mooncaks@gmail.com (P.-F.W.); jenny840209@gmail.com (J.-Y.G.); 3 Department of Ball Sports, University of Taipei, Taipei 111, Taiwan; rugby@go.utaipei.edu.tw; 4 Department of Physical Education, Shih Hsin University, Taipei 116, Taiwan; kuntien@contract.shu.edu.tw; 5 Office of Physical Education, Soochow University, Taipei 111002, Taiwan; lian@scu.edu.tw

**Keywords:** indigestible dextrin, TWK10, LP28, gut microbiota, synbiotics, HDL cholesterol

## Abstract

**Background:** Obesity is associated with metabolic dysregulation, mood disturbance, and poor sleep quality. Although dietary fiber and probiotic supplementation have both been proposed as microbiome-targeted strategies for obesity management, their independent and combined effects remain unclear. **Methods:** In this double-blind, randomized, placebo-controlled 2 × 2 factorial trial, 56 adults with obesity were randomized to placebo, dietary fiber, probiotic, or combined supplementation for 8 weeks. One withdrew during baseline assessment, and 55 participants were included in the intention-to-treat analysis. Outcomes included metabolic syndrome-related indicators, mood assessed by the Profile of Mood State, and sleep quality assessed by the Pittsburgh Sleep Quality Index. Intervention effects were evaluated using factorial ANCOVA with baseline adjustment. **Results:** No significant dietary fiber × probiotic interactions were detected. Dietary fiber supplementation showed selective favorable effects, mainly on HDL cholesterol and mood-related outcomes. Probiotic supplementation showed a significant main effect primarily on HDL cholesterol but did not remain significant after FDR correction. Sleep-related improvements were observed only in within-group analyses and were not supported by significant factorial ANCOVA effects. Combined supplementation did not provide additional benefits over single-component interventions. **Conclusions:** Dietary fiber supplementation may have selective favorable effects in adults with obesity, particularly on HDL cholesterol and mood-related outcomes. The absence of additional benefit from combined supplementation suggests that the effectiveness of synbiotic strategies may depend on the compatibility between the selected dietary fiber and probiotic strains.

## 1. Introduction

Obesity has become a major global health challenge, with its prevalence continuing to increase worldwide. Excess body fat adversely affects health and is strongly associated with chronic diseases [1]. There is a reciprocal interaction among obesity, diet, mood, and sleep. Obesity is closely linked to metabolic dysregulation, mood disturbance, and poor sleep quality, and these conditions may interact bidirectionally through changes in eating behavior, appetite regulation, stress, and sleep–wake patterns [2,3,4,5]. In addition, obesity increases the risk of related metabolic disorders. These risk factors are defined as metabolic syndrome, which affects about a quarter of adults in this world [6]. Metabolic syndrome (MetS) contains four central features: insulin resistance, obesity, atherogenic dyslipidemia and hypertension, which are interrelated and constitute the simplest comprehensive definition of metabolic syndrome [7]. Metabolic syndrome has a direct impact on health [8].

Gut microbiota are highly dynamic and can change rapidly in response to dietary shifts [9]. Because diet can greatly impact the host’s health by influencing the gut microbiota composition [10], manipulation of the gut biota through diet may be a promising approach for the prevention and treatment of metabolic syndrome and its associated disorders, including obesity [11]. Gut microbiota may contribute to obesity-related metabolic regulation, at least partly by influencing energy harvest from the diet [12,13]. In some cases of obesity, microbiota might be a causal factor and a therapeutic target for symptom improvement [14]. Moreover, there are the links between the microbial community and the development of obesity, cardiovascular disease, and metabolic syndromes [13]. Gut microbiome composition is associated with metabolic health, and gut biota can affect the risk of metabolic syndrome; it also affects the risk of depression through the synthesis of dopamine metabolites by intestinal flora. Preclinical studies suggest that intestinal microorganisms can affect psychiatric disorders, such as depression and anxiety. In fact, MetS is stronger than the link between obesity and depression [2].

Changes in gut microbiota composition and diversity are associated with obesity and weight gain, and several treatments such as prebiotics and probiotics, aimed at improving gut microbiome health, may be effective in preventing or treating obesity. Prebiotics indirectly modify gut microbiota composition by selectively promoting the growth of beneficial bacterial strains, thereby influencing microbial endocrine activity and host metabolism [15], whereas probiotics may exert their effects through strain-specific metabolic, immunomodulatory, and barrier-regulating mechanisms within the gut environment [16]. As the most valuable prebiotic, dietary fiber is composed of carbohydrates (monosaccharide polymers), includes resistant starch, non-starch polysaccharide, inulin and oligosaccharide such as fructooligosaccharides [17]. Inulin and inulin-type prebiotics can be fermented by colonic bacteria and stimulate the growth of beneficial genera such as Bifidobacterium [18], which are also commonly used as probiotic strains in clinical interventions. These prebiotics may influence the production of gastrointestinal hormones through the generation of short-chain fatty acids (SCFAs) [19,20,21], while probiotics may further modulate microbial metabolic activity and host responses depending on strain characteristics and host context [22]. Through these mechanisms, gut microbiota not only affect host metabolism but may also influence food intake and appetite regulation, thereby contributing to obesity management.

Although an increasing number of studies have investigated synbiotic (combined prebiotic and probiotic) interventions in obesity and metabolic disorders, important limitations remain. Many existing trials have focused on synbiotic supplementation without including appropriate single-component comparison groups, which restricts the ability to disentangle the independent effects of prebiotics and probiotics from their combined effects. Therefore, the aim of the present study was to systematically investigate the independent and combined effects of dietary fiber and probiotic supplementation on metabolic parameters, mood states, and sleep quality in adults with obesity.

## 2. Materials and Methods

### 2.1. Participants

Fifty-six eligible participants (height, 167.1 ± 7.3 cm; weight, 75.03 ± 17.44 kg; age, 41.1 ± 4.1 years) all voluntarily agreed to take the digitally provided, anonymous, online survey to enroll in this study. This was a single-center study conducted at the Laboratory of Exercise Biochemistry, Department of Sports Sciences, University of Taipei, Taipei, Taiwan. Participants were recruited from the local community through online advertisements and study announcements. The inclusion criteria were: (1) adults with obesity aged 30–45 years; and (2) body fat percentage >25% in men and >30% in women. Therefore, obesity eligibility in the present study was based on elevated body fat percentage (>25% in men and >30% in women) rather than BMI alone. Exclusion criteria included current medication use, menopause, and pregnancy. The procedures and purpose of the study, including the right to freely withdraw, were explained to the participants and their informed consent was obtained. This study was approved by Institutional Review Board of Taipei University (IRB-2021-006). Before the intervention, all participants were required to maintain their usual diet and not to take any additional supplements or drugs during the experiment.

### 2.2. Study Design

This study followed a double-blind, randomized, placebo-controlled design. Volunteers meeting the aforementioned criteria who gave informed consent were assigned, using a random-number table, to placebo (*n* = 14), dietary fiber (*n* = 14), probiotic (*n* = 14) and combined (*n* = 14) group. One participant in the dietary fiber group withdrew during baseline assessment before completing all baseline measurements. Therefore, 55 participants who completed baseline assessment and entered the intervention were included in the final analysis. Participants in all groups consumed placebo, fiber or probiotic supplements daily before breakfast for 8 weeks. To maintain participant blinding, all participants received both one capsule and powder sachets each day. The probiotic placebo consisted of maltodextrin-filled capsules, and the dietary fiber placebo consisted of maltodextrin powder. Thus, the supplement format was kept comparable across the four groups. Apart from the assigned supplementation, participants were asked to maintain their usual daily routines throughout the intervention period. Before the intervention, participants underwent anthropometric assessment, including height, weight, and whole-body composition analysis (dual-energy X-ray absorptiometry, DEXA) to confirm whether the whole-body fat percentage meets the test conditions. The tests were conducted at baseline and after intervention, including: (1) indicators of metabolic syndrome; (2) Profile of Mood State (POMS); and (3) Pittsburgh Sleep Quality Index (PSQI). Participants and outcome assessors were blinded to group allocation throughout the intervention and outcome assessments.

**Figure 1:**
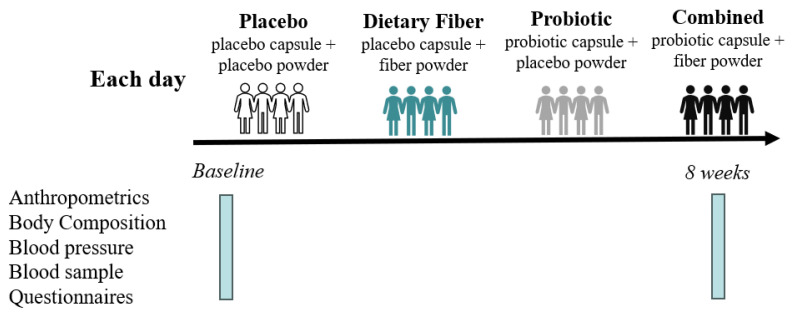
Study protocol and interventions.

The experimental flowchart is shown in Figure 1.

### 2.3. Trial Registration

This study was retrospectively registered in ClinicalTrials.gov under the registration number NCT06626867, 4 October 2024. Trial registration was completed after the study had been initiated because formal registration procedures were not completed before participant recruitment. The intervention protocol, outcome assessments, and assessment schedule were established before data analysis and were not modified according to the observed results.

### 2.4. Measurement

#### 2.4.1. Anthropometrics

Body height and weight were measured on a stadiometer (Takei Scientific Instruments, Tokyo, Japan) and digital scale (TANITA WB-110MA, Tokyo, Japan) to the nearest 0.1 cm and 0.1 kg without shoes and wearing light clothes. Waist circumference was measured at the midpoint between the iliac crest and the lowest rib margin, and hip circumference was assessed at the widest point around the buttocks. Based on the anthropometric metric measures, body mass index (BMI; kg/m^2^) and waist-to-hip ratio (WHR; waist/hip) were calculated.

#### 2.4.2. Body Composition

Body composition and bone mineral density were estimated by dual-energy X-ray absorptiometry (DEXA; GE Lunar Prodigy 8743, Madison, WI, USA). The DEXA results included body composition parameters, including fat-free mass, body fat percentage, fat mass, and bone mineral density. The same experienced technician performed the analysis of the scan.

#### 2.4.3. Blood Pressure

Blood pressure was measured from the non-dominant arm with a digital blood pressure monitor (Omron HEM-7121, OMRON Healthcare Co., Ltd., Kyoto, Japan) in sitting position after at least 15 min of rest.

#### 2.4.4. Biochemical Markers

Fasting blood samples were obtained in the morning after the participants had 12 h of overnight fasting. To obtain plasma or serum, the blood samples with 5 mL collected into blood sampling tubes with or without ethylene diamine tetraacetic acid (EDTA), respectively, were centrifuged at 3000 rpm at 4 °C for 15 min and then stored at −80 °C until analysis. The plasma blood samples were assessed using a fully automated analyzer (AU5800, Beckman Coulter^®^, Brea, CA, USA). A set of standard samples was collected and analyzed on the same day, including fasting glucose, high sensitivity C-reactive protein (HsCRP), fasting insulin, total cholesterol, low density lipoprotein (LDL-C), high density lipoprotein (HDL-C) and triglyceride (TG). Insulin was measured by using electrochemiluminescence immunoassay (ECLIA) (Cobas e801, Roche Diagnostics, Mannheim, Germany); glycated hemoglobin (HbA1c) was measured by using an ion exchange resin composed of hydrophilic polymer of methacrylate ester copolymer (HA-8180V, Arkray, Kyoto, Japan).

#### 2.4.5. Questionnaires

Participants completed two questionnaires: (1) the Abbreviated Profile of Mood State [23], revised from the Profile of Mood State (POMS) [24], and (2) the Chinese version of the Pittsburgh Sleep Quality Index [25], translated from the Pittsburgh Sleep Quality Index (PSQI) [26].

### 2.5. Supplements Intervention

Supplements were taken daily before breakfast. Dietary fiber was indigestible dextrin (The WiseMan’s Dining; King Car OTSUKA Co., Taipei, Taiwan), 6 g per sachet, 3 sachets/day (18 g/day). Probiotics were provided as a multi-strain capsule (Power Probiotics Fit XS; Power Probiotics Health Management Consulting Co., Taipei, Taiwan) containing *Lactobacillus plantarum* TWK10 and LP28, *Bifidobacterium longum*, *Lactobacillus rhamnosus*, and *Streptococcus thermophilus* (2 × 10^10^ CFU/capsule), 1 capsule/day. placebos contained maltodextrin (colored capsule) and maltodextrin powder (5 g/day). The placebo capsule and placebo powder were used to match the probiotic capsule and dietary fiber powder, respectively, so that all participants received the same supplement format. For blinding, all participants received one capsule plus one powder daily: Placebo (placebo capsule + placebo powder), dietary fiber (placebo capsule + fiber powder), probiotic (probiotic capsule + placebo powder), and combined (probiotic capsule + fiber powder). The selected doses were based on the commercially recommended intake and previous human supplementation studies using indigestible dextrin and multi-strain probiotics.

### 2.6. Statistical Analysis

All statistical analyses were performed using R software version 4.2.2 (R Core Team, R Foundation for Statistical Computing, Vienna, Austria) in RStudio. All tests were two-sided, and *p* < 0.05 was considered statistically significant. Analyses were performed according to the intention-to-treat principle. Given the small amount of missing data (<5%) and the relatively small sample size, baseline values for participants lost to follow-up were carried forward as post-intervention values [27,28].

Data distribution and homogeneity of variance were assessed using the Shapiro–Wilk test and Levene’s test, respectively. Descriptive statistics are presented as mean ± SD. Within-group changes from baseline to post-intervention were assessed separately for each intervention group. Normally distributed variables were analyzed using paired-samples *t* tests, whereas non-normally distributed variables were analyzed using Wilcoxon signed-rank tests.

The intervention effects on metabolic syndrome-related features, mood disturbance, and sleep quality were evaluated using 2 × 2 factorial analysis of covariance (ANCOVA). For each post-intervention outcome, dietary fiber supplementation (yes/no), probiotic supplementation (yes/no), and their interaction were entered as fixed factors, with the corresponding baseline value included as a mean-centered covariate. The dietary fiber × probiotic interaction was tested first. When the interaction term was not statistically significant, the final model was refitted without the interaction term to estimate the independent main effects of dietary fiber and probiotic supplementation.

Factorial ANCOVA results are reported with effect sizes as partial eta-squared (η^2^p). Model-based estimates are presented as adjusted marginal means and between-factor mean differences with 95% confidence intervals where available. To account for multiplicity across related outcomes, Benjamini–Hochberg false discovery rate (FDR) correction was applied separately within prespecified outcome domains. These domains comprised obesity-related indicators (BMI, body fat percentage, fat mass, muscle mass, bone mass, waist circumference, hip circumference, and waist-to-hip ratio), dyslipidemia-related indicators (HDL cholesterol, LDL cholesterol, and triglycerides), glycemic indicators (fasting glucose and HbA1c), blood pressure indicators (systolic and diastolic blood pressure), mood outcomes (POMS subscales and total mood disturbance), and sleep outcomes (PSQI component scores and global PSQI score). Findings that remained significant after FDR correction were interpreted as statistically robust, whereas nominally significant findings that did not survive FDR correction were interpreted as exploratory.

## 3. Results

### 3.1. Participant Characteristics at Baseline

A total of 56 participants were randomized to the placebo (*n* = 14), dietary fiber (*n* = 14), probiotic (*n* = 14), and combined supplementation (*n* = 14) groups. One participant in the dietary fiber group withdrew during baseline assessment before completing all baseline measurements. Therefore, 55 participants who completed baseline assessment and entered the intervention were included in the intention-to-treat analysis. Of these participants, 26 were men and 29 were women. Baseline characteristics were comparable across the four groups, with no significant between-group differences observed in age, height, body weight, BMI, or body fat percentage (all *p* > 0.05; Table 1). The study flow and intervention timeline are shown in Figure 1. No serious adverse events were reported during the intervention. One participant in the dietary fiber group did not provide post-intervention data for personal reasons.

### 3.2. Effects on Key Features Related to Metabolic Syndrome

In the factorial ANCOVA models, no significant fiber × probiotic interactions were detected for metabolic outcomes; therefore, the main effects of dietary fiber and probiotic supplementation were examined (Table 2 and Table 3). Dietary fiber supplementation showed a significant main effect on HDL-C that remained significant after FDR correction. The adjusted post-intervention HDL-C level was higher in the fiber group than in the no-fiber group (*p* = 0.009, FDR-adjusted *p* = 0.027, η^2^p = 0.145). Dietary fiber also showed an uncorrected significant effect on diastolic blood pressure (*p* = 0.038, FDR-adjusted *p* = 0.075, η^2^p = 0.091), but this effect did not survive FDR correction. No significant effects of dietary fiber were observed for the remaining metabolic outcomes. Probiotic supplementation showed an uncorrected significant main effect on HDL-C (*p* = 0.036, FDR-adjusted *p* = 0.109, η^2^p = 0.096), but this effect did not remain significant after FDR correction. No significant main effects of probiotic supplementation were detected for the other obesity-related, lipid, blood pressure, or glycemic outcomes.

**Figure 2:**
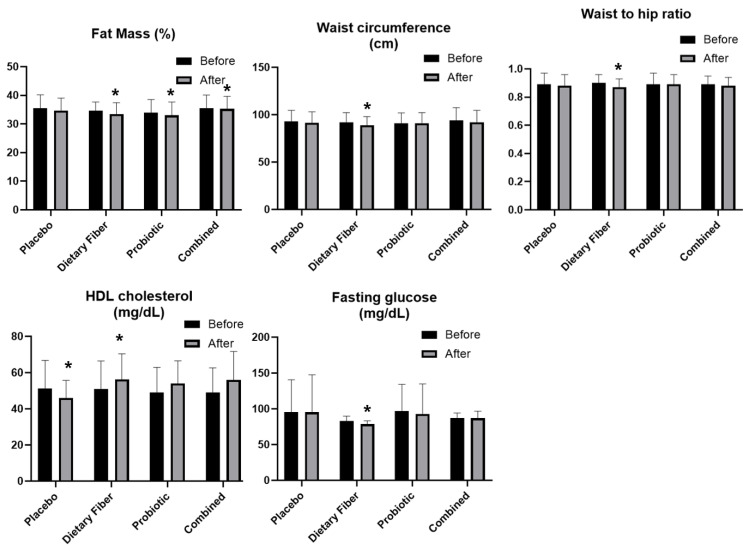
Fat mass, waist circumference, waist-to-hip ratio, HDL cholesterol and fasting glucose in participants with placebo group, dietary fiber, probiotic and combined at baseline and after 8 weeks. * Exploratory within-group pre–post difference compared with baseline (*p* < 0.05). Values are expressed as mean ± SD.

Exploratory within-group analyses demonstrated selective improvements in metabolic parameters following the 8-week intervention (Figure 2; Supplementary Table S1). Body fat percentage decreased significantly in the dietary fiber and probiotic groups, while a smaller but significant reduction was also observed in the combined group. Waist circumference and waist-to-hip ratio decreased significantly only in the dietary fiber group. Fasting glucose levels were significantly reduced in the dietary fiber group, whereas no significant within-group changes were observed in HbA1c or BMI across groups. HDL cholesterol decreased in the placebo group but increased significantly in the dietary fiber group, while changes in LDL cholesterol, triglycerides, and blood pressure were not statistically significant within individual groups.

### 3.3. Effects on Mood Disturbance

In the factorial ANCOVA models, dietary fiber supplementation showed significant main effects on total mood disturbance and several mood subscales after FDR correction (Table 4). Compared with participants not receiving dietary fiber, those receiving dietary fiber had a lower adjusted total mood disturbance score (*p* = 0.003, FDR-adjusted *p* = 0.005, η^2^p = 0.168).

Significant main effects of dietary fiber were also observed for confusion (*p* < 0.001, FDR-adjusted *p* = 0.003, η^2^p = 0.227), fatigue (*p* = 0.032, FDR-adjusted *p* = 0.043, η^2^p = 0.089), anger (*p* = 0.004, FDR-adjusted *p* = 0.006, η^2^p = 0.154), tension (*p* = 0.001, FDR-adjusted *p* = 0.005, η^2^p = 0.188), and depression (*p* = 0.003, FDR-adjusted *p* = 0.005, η^2^p = 0.168). No significant main effects of dietary fiber were observed for vigor or esteem. Probiotic supplementation did not show significant main effects on total mood disturbance or any mood subscale after FDR correction (Table 5). The adjusted mean differences between the probiotic and no-probiotic groups were not statistically significant for total mood disturbance, confusion, fatigue, anger, tension, depression, vigor, or esteem.

**Figure 3:**
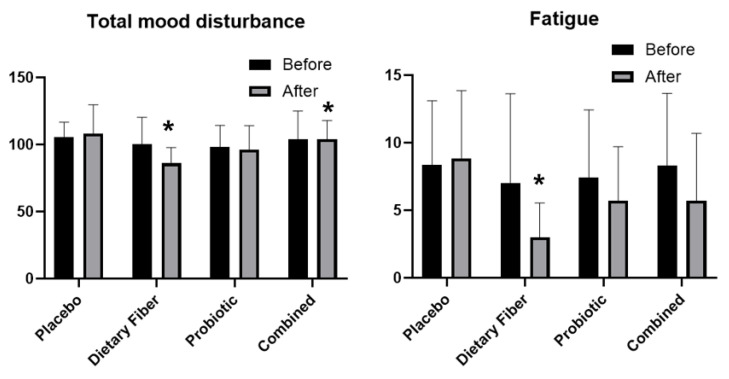
Total mood disturbance and fatigue in participants with placebo group, dietary fiber, probiotic and combined at baseline and after 8 weeks. * Exploratory within-group pre–post difference compared with baseline (*p* < 0.05). Values are expressed as mean ± SD.

Exploratory within-group analyses indicated a significant reduction in total mood disturbance (TMD) in the dietary fiber and combined groups, whereas no significant change was observed in the placebo or probiotic groups (Figure 3; Supplementary Table S2). Several negative mood subscales, including confusion and fatigue, decreased significantly in the dietary fiber group, while confusion increased in the placebo group. Positive mood domains, including vigor and esteem, increased significantly in the placebo group, with smaller or non-significant changes observed in the intervention groups.

### 3.4. Effects on Sleep Quality

In the factorial ANCOVA models, neither dietary fiber nor probiotic supplementation showed significant main effects on sleep quality outcomes after FDR correction (Table 6 and Table 7). For dietary fiber, no significant differences were observed in global PSQI score or in any individual PSQI component, including subjective sleep quality, sleep latency, sleep duration, habitual sleep efficiency, sleep disturbance, use of sleep medication, and daytime dysfunction. Similarly, probiotic supplementation had no significant effects on global PSQI score or any PSQI component.

**Figure 4:**
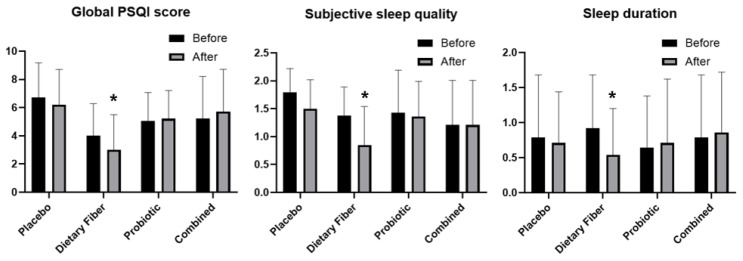
Global PSQI score, subjective sleep quality and sleep disturbance in participants with placebo group, dietary fiber, probiotic and combined at baseline and after 8 weeks. * Exploratory within-group pre–post difference compared with baseline (*p* < 0.05). Values are expressed as mean ± SD.

Sleep quality outcomes assessed by the PSQI are summarized in Figure 4 and Supplementary Table S3. Exploratory within-group analyses revealed a significant reduction in global PSQI score in the dietary fiber group, accompanied by improvements in subjective sleep quality and sleep duration. No significant within-group changes were observed in the placebo, probiotic, or combined groups.

## 4. Discussion

The present study used a double-blind, randomized, placebo-controlled 2 × 2 factorial design to investigate the independent and combined effects of dietary fiber and probiotic supplementation on metabolic syndrome-related features, mood disturbance, and sleep quality in adults with obesity. The interpretation of the present findings was based primarily on the baseline-adjusted factorial ANCOVA models, whereas within-group pre–post changes were considered exploratory and descriptive. Overall, dietary fiber showed a more consistent effect on mood-related outcomes, while its metabolic effects were mainly limited to HDL cholesterol after FDR correction. The effects on sleep quality were not statistically significant in the factorial ANCOVA models. Importantly, no significant interaction between dietary fiber and probiotic supplementation was observed, suggesting that the combined intervention did not show clear additional benefits under the present study conditions.

### 4.1. Effects on Key Features Related to Metabolic Syndrome

The present study suggests that dietary fiber supplementation may selectively improve HDL cholesterol in adults with obesity. After FDR correction, HDL-C was the only metabolic syndrome-related outcome that remained statistically significant for dietary fiber. Although dietary fiber was associated with lower diastolic blood pressure before correction, this finding did not survive FDR correction and should therefore be interpreted as exploratory. Similarly, probiotic supplementation showed an uncorrected association with higher HDL-C, but this effect was no longer significant after FDR correction. Prebiotics may influence key features of metabolic syndrome, such as blood glucose homeostasis and dyslipidemia [29]. In the present study, fasting glucose was not significantly affected by dietary fiber, which is consistent with most previous studies [30,31,32,33]. Dietary fiber intake has also been associated with a reduction in waist circumference [34]. However, the effects of prebiotics on lipoproteins remain inconsistent. Differences in prebiotic type, dose, intervention duration, and participant characteristics may partly explain these inconsistent findings. A meta-analysis of 20 controlled clinical studies reported in 14 articles found that resistant starch supplementation was associated with a significant reduction in LDL cholesterol, but not HDL cholesterol [35]. Another study reported no changes in LDL cholesterol or triglycerides [29]. In addition, the study found a decrease in HDL cholesterol after dietary fiber supplementation [36], which contrasts with our findings. In the present study, HDL cholesterol appeared to be the most responsive lipid-related outcome.

Several mechanisms may account for these findings. Dietary fiber may influence metabolic regulation through colonic fermentation and the associated changes in gut microbial activity [35]. Fermentation-derived metabolites, particularly short-chain fatty acids, may in turn contribute to the regulation of lipid and glucose metabolism. In addition, certain types of fiber may increase intestinal viscosity, thereby reducing the absorption of triglycerides and cholesterol [37]. The findings of the present study suggest that the effects of dietary fiber are unlikely to be uniform and may depend on the specific type and dose of fiber used. This may also help explain why combined supplementation did not produce broader metabolic benefits, as the effectiveness of such interventions may depend on whether the selected fiber can provide a suitable substrate for the probiotic strains used.

Diastolic blood pressure showed a nominal reduction with dietary fiber supplementation, but this effect did not remain significant after FDR correction. Therefore, this finding should be interpreted cautiously and considered hypothesis-generating. A systematic review and meta-analysis showed that the effects of dietary fiber on blood pressure may vary according to fiber type [38], while another study reported a significant reduction in systolic blood pressure but not in diastolic blood pressure after psyllium supplementation [39]. These findings suggest that blood pressure responses to dietary fiber are not uniform and may depend on the characteristics of the fiber administered. A recent study has also suggested that higher dietary fiber intake may support blood pressure control, potentially through gut microbiota-related pathways and the production of short-chain fatty acids [40]. One possible mechanism is that short-chain fatty acids produced during fiber fermentation may interact with host receptors involved in vascular tone, inflammation, and sodium handling, thereby contributing to blood pressure regulation [41]. Taken together, these findings suggest that the beneficial effect of dietary fiber on blood pressure may be influenced by the type, dose, and intervention duration of dietary fiber.

### 4.2. Effects on Mood Disturbance

The effects on mood disturbance were more pronounced than those observed for most metabolic outcomes. In the present study, dietary fiber supplementation was associated with significant reductions in total mood disturbance and improvements in several negative mood dimensions, including confusion, fatigue, anger, tension, and depression. These findings remained significant after FDR correction, suggesting that the mood-related effects were more consistent than the metabolic or sleep-related outcomes. Similar findings have also been reported in previous studies, in which resistant starch supplementation improved depression, anxiety, and stress in some populations [42,43,44]. However, not all studies have shown consistent effects, and some have reported no significant change in depression-related outcomes [45,46]. These differences may be related to the study population, intervention duration, and the type of dietary fiber used. Fermentable fibers may alter gut microbial activity and thereby influence gut–brain communication, which could contribute to improvements in emotional well-being [47,48]. At the same time, the consistent improvement across multiple negative mood dimensions in the present study suggests that the psychological effects of dietary fiber may not be limited to a single aspect of mood. In contrast, probiotic supplementation alone did not show significant main effects on total mood disturbance or any individual mood subscale. This suggests that, under the present intervention conditions, dietary fiber may have played a more prominent role than probiotics in influencing mood-related outcomes, although this result should be interpreted cautiously given the relatively small sample size and short intervention period. In addition, because mood disturbance was assessed using a self-reported questionnaire, the observed improvements may not be attributable solely to dietary fiber supplementation. Expectancy effects, placebo effects, increased health awareness, repeated contact with researchers, and the psychological impact of participating in a structured intervention study may also have contributed to the POMS improvements.

### 4.3. Effects on Sleep Quality

The effects on sleep quality were more limited than those observed for mood disturbance. In the present study, dietary fiber supplementation was associated with improvements in sleep-related outcomes in the within-group analysis, particularly in global PSQI score, subjective sleep quality, and sleep duration, whereas no significant main effects were observed in the factorial model. Therefore, the evidence for sleep-related benefits was weak, and these within-group findings should be interpreted as exploratory and descriptive. Similar findings have been reported in previous studies, in which prebiotic supplementation improved sleep-related outcomes in some populations [49,50,51,52]. Poor sleep quality is closely related to fatigue, daytime sleepiness, mood disturbance, and reduced function [53], and improvement in these symptoms may partly contribute to better sleep quality. Sleep may also be influenced by gut microbial activity and related metabolites, which may partly explain the potential effect of dietary fiber on sleep [50]. In contrast, probiotic supplementation alone did not show significant effects on sleep-related outcomes. Overall, these findings suggest that dietary fiber may have potential benefits for sleep, although this interpretation should be made cautiously.

### 4.4. Independent Versus Combined Effects

An important strength of the present study is the 2 × 2 factorial design, which addresses a common limitation of many previous synbiotic studies, where combined interventions were tested without appropriate single-component comparison groups. In the present study, no significant interaction between dietary fiber and probiotic supplementation was observed, suggesting that the combined intervention did not provide additional benefit beyond the effects of each component alone during the intervention period. Our findings differ from those of previous synbiotic studies that reported stronger effects with combined supplementation [54]. In particular, a recent systematic review of randomized clinical trials found that synbiotics showed more pronounced benefits than probiotics or prebiotics alone for insulin resistance, fasting glucose, fasting insulin, LDL cholesterol, triglycerides, and HDL cholesterol in overweight and obese women [55]. In addition, broader reviews of microbiome-targeted interventions have suggested that probiotic and synbiotic approaches may be among the more consistent strategies for improving glucose and lipid metabolism, although these effects appear to vary by population, intervention duration, and formulation [56]. Rather than indicating that synbiotic strategies are ineffective, the present results suggest that the effect of a combined intervention may depend partly on whether the selected prebiotic is actually suitable for the probiotic strains used. In this sense, choosing an appropriate prebiotic may be just as important as choosing the probiotic itself.

### 4.5. Strengths and Limitations

This study has several limitations that should be acknowledged. First, this trial was retrospectively registered, and the sample size was informed by a previous similar intervention study rather than by a separate formal a priori power calculation for the present trial, which may have reduced outcome reporting transparency and made it difficult to determine whether the study was adequately powered for all primary outcomes. The relatively small sample size, with only 13–14 participants in each group, may also have limited the statistical power to detect effects for outcomes with substantial inter-individual variability. Therefore, both significant and non-significant findings should be interpreted cautiously, and the present results should be considered preliminary. Second, the intervention period was limited to 8 weeks and may not have been long enough to observe more stable changes in body composition, glycemic markers, or sleep quality. Third, although the intervention was conceptually linked to the gut microbiota, no direct measurements of the microbiome, metabolites such as short-chain fatty acids, or inflammatory markers were included. Therefore, all microbiome-related mechanistic explanations should be regarded as speculative and hypothesis-generating. Fourth, dietary intake and physical activity were not systematically monitored throughout the intervention, and changes in these behaviors may have influenced metabolic, mood-related, or sleep-related outcomes. Fifth, sleep apnea was not systematically assessed, which may have affected the interpretation of PSQI-based sleep outcomes. Future studies should screen for or exclude clinically relevant obstructive sleep apnea to better isolate the effects of supplementation on sleep quality. In addition, although maltodextrin was used as a practical placebo to match the supplement format, it may not be completely metabolically inert. Its glycemic properties could have attenuated between-group differences, particularly for glycemic and lipid-related outcomes. Finally, the study population consisted of adults with obesity defined by elevated body fat percentage within a relatively specific demographic range, which may limit the generalizability to individuals with BMI-defined obesity or more severe obesity. Despite these limitations, the present study suggests that dietary fiber supplementation may have broader benefits than probiotic supplementation alone in adults with obesity. In particular, dietary fiber was associated with favorable changes in selected metabolic syndrome-related features and more consistent improvements in mood-related outcomes, whereas probiotic supplementation showed a narrower effect profile, mainly limited to HDL cholesterol. From a practical perspective, these findings support the potential value of dietary fiber as an accessible nutritional strategy for obesity management, especially when psychological well-being is also a concern, but larger and longer trials with dietary monitoring and direct mechanistic measures are needed before firm clinical conclusions can be drawn.

## 5. Conclusions

The present study suggests that dietary fiber supplementation may provide broader benefits than probiotic supplementation alone in adults with obesity, particularly for selected metabolic syndrome-related features and mood disturbance. Improvements in sleep-related outcomes were observed only in the within-group analysis, and no additional benefit of combined supplementation was observed, highlighting that the effectiveness of a combined intervention may depend on the compatibility between the selected dietary fiber and probiotic strains. Further studies with larger sample sizes, longer intervention durations, and direct mechanistic measures are needed.

## Figures and Tables

**Table 1:** Backgrounds of participants.

Characteristics	Total (*n* = 55)	Placebo (*n* = 14)	Dietary Fiber (*n* = 13)	Probiotic (*n* = 14)	Combined (*n* = 14)	*p*-Value
Male (*n*; %)	26 (47.3%)	7 (50.0%)	6 (46.2%)	7 (50.0%)	6 (42.9%)	0.978
Age (years)	41.1 ± 4.1	41.1 ± 3.6	42.5 ± 3.9	40.6 ± 4.4	40.2 ± 4.6	0.482
BMI (kg/m^2^)	26.65 ± 4.56	26.86 ± 4.58	26.00 ± 3.75	26.87 ± 4.81	26.85 ± 5.35	0.953
Fat mass (%)	35.11 ± 4.32	35.58 ± 4.62	34.65 ± 3.89	34.68 ± 4.54	35.52 ± 4.57	0.906
Male (%)	33.18 ± 3.78	33.34 ± 2.83	32.87 ± 3.17	33.19 ± 5.09	33.32 ± 4.54	0.997
Female (%)	36.84 ± 4.09	37.81 ± 5.15	36.17 ± 4.01	36.17 ± 3.69	37.17 ± 4.10	0.859

Data presented as mean ± SD.

**Table 2:** Main effect of dietary fiber on key features of metabolic syndrome.

Outcome	Fiber Adj. Mean (95% CI)	No Fiber Adj. Mean (95% CI)	Mean Difference (Fiber − No Fiber, 95% CI)	F	*p*	*p* FDR	η^2^p
BMI (kg/m^2^)	26.47 (26.30, 26.80)	26.51 (26.22, 26.81)	−0.04 (−0.38, 0.30)	0.11	0.737	0.814	0.002
Body fat (%)	34.14 (33.40, 34.90)	33.91 (33.24, 34.59)	−0.25 (−1.30, 0.80)	0.26	0.614	0.814	0.005
Waist circumference (cm)	90.80 (89.50, 92.10)	92.07 (90.78, 93.36)	−1.27 (−3.04, 0.50)	1.90	0.175	0.699	0.040
Hip circumference (cm)	104.0 (103.0, 105.0)	103.0 (102.0, 104.0)	−0.60 (−1.74, 0.54)	1.17	0.285	0.762	0.025
Waist-to-hip ratio	0.874 (0.862, 0.887)	0.890 (0.878, 0.902)	−0.016 (−0.033, 0.001)	3.30	0.076	0.607	0.067
HDL-C (mg/dL)	57.50 (53.60, 61.40)	49.80 (45.90, 53.70)	7.74 (2.20, 13.27)	7.47	0.009	0.027 *	0.145
LDL-C (mg/dL)	120.0 (112.2, 127.8)	120.2 (111.4, 128.9)	−0.20 (−12.21, 12.60)	0.01	0.939	0.939	<0.001
Triglycerides (mg/dL)	95.80 (67.77, 123.8)	122.7 (95.18, 150.2)	−26.9 (−66.7, 12.8)	2.10	0.155	0.222	0.045
SBP (mmHg)	117.5 (112.9, 122.2)	118.8 (113.9, 123.7)	−1.29 (−7.85, 5.26)	0.21	0.647	0.647	0.005
DBP (mmHg) ^†^	75.00 (71.26, 78.78)	80.45 (76.84, 84.06)	−5.42 (−10.64, −0.20)	4.58	0.038	0.075	0.091
Fasting glucose (mg/dL)	86.40 (74.36, 98.44)	90.24 (78.32, 102.2)	−3.84 (−20.91, 13.23)	0.18	0.675	0.675	0.004
HbA1c (%)	5.63 (5.53, 5.73)	5.69 (5.59, 5.79)	−0.06 (−0.20, 0.08)	0.68	0.416	0.675	0.015

Values are adjusted means with 95% confidence intervals (CI), estimated by 2 × 2 factorial ANCOVA with mean-centered baseline as covariate. * *p* FDR < 0.05 (survives FDR correction). ^†^ *p* < 0.05 (uncorrected) but *p* FDR ≥ 0.05. η^2^p = partial eta-squared.

**Table 3:** Main effect of probiotic on key features of metabolic syndrome.

Outcome	Probiotic Adj. Mean (95% CI)	No Probiotic Adj. Mean (95% CI)	Mean Difference (Probiotic − No Probiotic, 95% CI)	F	*p*	*p* FDR	η^2^p
BMI (kg/m^2^)	26.57 (26.31, 26.83)	26.44 (26.15, 26.73)	0.13 (−0.21, 0.48)	0.57	0.455	0.776	0.012
Body fat (%)	34.03 (33.24, 34.73)	33.96 (33.17, 34.76)	−0.07 (−1.12, 0.98)	0.05	0.831	0.965	0.001
Waist circumference (cm)	92.04 (90.84, 93.24)	90.90 (89.57, 92.23)	1.08 (−0.68, 2.85)	1.47	0.232	0.776	0.031
Hip circumference (cm)	104.0 (103.0, 105.0)	103.0 (102.0, 104.0)	0.46 (−0.68, 1.60)	0.65	0.426	0.776	0.014
Waist-to-hip ratio	0.886 (0.874, 0.897)	0.879 (0.867, 0.892)	0.006 (−0.011, 0.023)	0.50	0.485	0.776	0.011
HDL-C (mg/dL) ^†^	56.60 (52.80, 60.40)	50.68 (46.62, 54.74)	5.90 (0.35, 11.47)	4.66	0.036	0.109	0.096
LDL-C (mg/dL)	122.0 (114.0, 131.0)	118.0 (109.0, 127.0)	3.94 (−8.50, 16.4)	0.40	0.530	0.530	0.009
Triglycerides (mg/dL)	122.0 (94.60, 148.0)	96.90 (68.60, 125.0)	24.6 (−15.0, 64.2)	1.55	0.220	0.330	0.034
SBP (mmHg)	119.0 (114.0, 123.0)	118.0 (113.0, 122.0)	1.26 (−5.25, 7.76)	0.17	0.685	0.913	0.004
DBP (mmHg)	77.60 (74.07, 81.20)	77.84 (74.04, 81.70)	−0.20 (−5.43, 5.02)	0.00	0.947	0.947	<0.001
Fasting glucose (mg/dL)	89.10 (77.70, 101.0)	87.50 (75.30, 99.60)	1.70 (−15.0, 18.4)	0.04	0.845	0.965	<0.001
HbA1c (%)	5.64 (5.54, 5.73)	5.69 (5.59, 5.79)	−0.05 (−0.19, 0.09)	0.52	0.475	0.845	0.012

Values are adjusted means with 95% confidence intervals (CI), estimated by 2 × 2 factorial ANCOVA with mean-centered baseline as covariate. ^†^ *p* < 0.05 (uncorrected) but *p* FDR ≥ 0.05. η^2^p = partial eta-squared.

**Table 4:** Main effect of dietary fiber on key features of mood disturbance.

Outcome	Fiber Adj. Mean (95% CI)	No Fiber Adj. Mean (95% CI)	Mean Difference (Fiber − No Fiber, 95% CI)	F	*p*	*p* FDR	η^2^p
Total mood disturbance	88.55 (82.54, 94.56)	102.2 (96.10, 108.4)	−13.5 (−21.9, −5.00)	10.07	0.003	0.005 *	0.168
Confusion	2.11 (0.40, 3.81)	6.73 (5.05, 8.40)	−4.62 (−7.01, −2.23)	14.68	<0.001	0.003 *	0.227
Fatigue	4.74 (3.10, 6.37)	7.29 (5.69, 8.90)	−2.56 (−4.85, −0.27)	4.86	0.032	0.043 *	0.089
Anger	2.23 (0.79, 3.67)	5.30 (3.88, 6.72)	−3.07 (−5.10, −1.04)	9.12	0.004	0.006 *	0.154
Tension	1.49 (0.66, 2.33)	3.49 (2.67, 4.31)	−2.00 (−3.18, −0.83)	11.55	0.001	0.005 *	0.188
Depression	0.55 (−0.10, 1.21)	2.00 (1.36, 2.65)	−1.45 (−2.37, −0.53)	10.07	0.003	0.005 *	0.168
Vigor	13.13 (11.50, 14.76)	13.90 (12.30, 15.50)	−0.73 (−3.04, 1.58)	0.38	0.541	0.609	0.008
Esteem	9.34 (8.31, 10.37)	8.99 (7.98, 9.99)	0.35 (−1.09, 1.79)	0.27	0.609	0.609	0.005

Values are adjusted means with 95% confidence intervals (CI), estimated by 2 × 2 factorial ANCOVA with mean-centered baseline as covariate. * *p* FDR < 0.05 (survives FDR correction). η^2^p = partial eta-squared.

**Table 5:** Main effect of probiotic on key features of mood disturbance.

Outcome	Probiotic Adj. Mean (95% CI)	No Probiotic Adj. Mean (95% CI)	Mean Difference (Probiotic − No Probiotic, 95% CI)	F	*p*	*p* FDR	η^2^p
Total mood disturbance	93.46 (87.56, 99.50)	97.03 (90.95, 103.1)	−3.51 (−12.0, 4.95)	0.72	0.401	0.591	0.014
Confusion	3.62 (1.94, 5.29)	5.21 (3.51, 6.92)	−1.60 (−3.99, 0.80)	1.91	0.173	0.591	0.037
Fatigue	5.73 (4.13, 7.33)	6.30 (4.67, 7.94)	−0.57 (−2.86, 1.72)	0.29	0.593	0.678	0.006
Anger	3.29 (1.88, 4.71)	4.23 (2.79, 5.68)	−0.94 (−2.96, 1.08)	0.95	0.334	0.591	0.019
Tension	2.18 (1.36, 3.00)	2.80 (1.97, 3.64)	−0.62 (−1.79, 0.54)	1.20	0.279	0.591	0.023
Depression	1.02 (0.38, 1.66)	1.54 (0.88, 2.19)	−0.51 (−1.43, 0.40)	1.33	0.255	0.591	0.026
Vigor	13.39 (11.80, 15.00)	13.57 (12.01, 15.27)	−0.23 (−2.57, 2.10)	0.05	0.832	0.832	<0.001
Esteem	8.89 (7.88, 9.90)	9.43 (8.40, 10.46)	−0.54 (−1.99, 0.91)	0.60	0.443	0.591	0.012

Values are adjusted means with 95% confidence intervals (CI), estimated by 2 × 2 factorial ANCOVA with mean-centered baseline as covariate.

**Table 6:** Main effect of dietary fiber on key features of sleep quality.

Outcome	Fiber Adj. Mean (95% CI)	No Fiber Adj. Mean (95% CI)	Mean Difference (Fiber − No Fiber, 95% CI)	F	*p*	*p* FDR	η^2^p
Global PSQI score	4.93 (4.00, 5.86)	5.46 (4.55, 6.38)	−0.53 (−1.85, 0.78)	0.56	0.457	0.834	0.011
Subjective sleep quality	1.10 (0.86, 1.34)	1.36 (1.13, 1.59)	−0.26 (−0.60, 0.08)	2.24	0.141	0.488	0.043
Sleep latency	0.76 (0.53, 1.00)	0.87 (0.64, 1.10)	−0.10 (−0.44, 0.23)	0.40	0.529	0.834	0.008
Sleep duration	0.66 (0.40, 0.92)	0.75 (0.50, 1.01)	−0.09 (−0.46, 0.27)	0.24	0.625	0.834	0.005
Habitual sleep efficiency	0.48 (0.16, 0.79)	0.43 (0.11, 0.74)	0.05 (−0.40, 0.50)	0.06	0.810	0.926	0.001
Sleep disturbance	1.16 (1.04, 1.28)	1.05 (0.94, 1.17)	0.11 (−0.06, 0.28)	1.82	0.183	0.488	0.035
Use of sleep medication	0.20 (−0.01, 0.41)	0.20 (−0.01, 0.40)	0.01 (−0.29, 0.29)	0.005	0.943	0.943	<0.001
Daytime dysfunction	0.54 (0.30, 0.79)	0.83 (0.59, 1.07)	−0.29 (−0.63, 0.06)	2.67	0.109	0.488	0.051

Values are adjusted means with 95% confidence intervals (CI), estimated by 2 × 2 factorial ANCOVA with mean-centered baseline as covariate.

**Table 7:** Main effect of probiotic on key features of sleep quality.

Outcome	Probiotic Adj. Mean (95% CI)	No Probiotic Adj. Mean (95% CI)	Mean Difference (Probiotic − No Probiotic, 95% CI)	F	*p*	*p* FDR	η^2^p
Global PSQI score	5.68 (4.77, 6.60)	4.71 (3.78, 5.64)	0.97 (−0.34, 2.28)	2.24	0.141	0.553	0.043
Subjective sleep quality	1.35 (1.11, 1.58)	1.11 (0.88, 1.35)	0.23 (−0.11, 0.57)	1.87	0.177	0.553	0.036
Sleep latency	0.83 (0.60, 1.07)	0.80 (0.56, 1.04)	0.03 (−0.30, 0.37)	0.04	0.851	0.860	<0.001
Sleep duration	0.82 (0.57, 1.08)	0.59 (0.33, 0.85)	0.23 (−0.13, 0.60)	1.63	0.207	0.553	0.032
Habitual sleep efficiency	0.58 (0.26, 0.89)	0.33 (0.01, 0.65)	0.25 (−0.20, 0.70)	1.21	0.276	0.553	0.024
Sleep disturbance	1.13 (1.02, 1.25)	1.08 (0.97, 1.20)	0.05 (−0.12, 0.21)	0.33	0.569	0.860	0.007
Use of sleep medication	0.22 (0.01, 0.42)	0.18 (−0.03, 0.39)	0.04 (−0.25, 0.33)	0.05	0.817	0.860	0.001
Daytime dysfunction	0.70 (0.47, 0.94)	0.67 (0.43, 0.91)	0.04 (−0.31, 0.38)	0.03	0.860	0.860	<0.001

Values are adjusted means with 95% confidence intervals (CI), estimated by 2 × 2 factorial ANCOVA with mean-centered baseline as covariate.

## Data Availability

The data presented in this study are available on request from the corresponding author due to privacy protection and ethical restrictions related to participant data.

## Supplementary Materials

The following supporting information can be downloaded at: https://www.mdpi.com/article/10.3390/nu18121851/s1, Table S1: Key features of metabolic syndrome before and after the intervention by group; Table S2: Mood disturbance before and after the intervention by group; Table S3: Sleep quality before and after the intervention by group.
