# Supplementary material for: Effects of Probiotic and Dietary Fiber Supplementation on Metabolic Syndrome-Related Features, Mood, and Sleep in Adults with Obesity
Source: Nutrients. 2026 Jun 9;18(12):1851. doi: 10.3390/nu18121851 (PMC13306066; doi:10.3390/nu18121851)
Supplement: Supplementary file 1 [file nutrients-18-01851-s001.zip › nutrients-4283386-supplementary.pdf]

Supplementary Table S1. Key features of metabolic syndrome before and after the intervention by group.

	Placebo (n =14)	Dietary fiber (n =13)	Probiotic (n =14)	Combined (n = 14)
Fat mass (%)				
Before	35.58 ± 4.62	34.65 ± 3.89	34.68 ± 4.54	35.52 ± 4.57
After	34.74 ± 4.34	33.45 ± 4.84*	33.06 ± 4.70*	35.33 ± 4.33*
Waist circumference				
Before	92.83 ± 11.90	92.13 ± 10.21	91.51 ± 10.95	93.91 ± 13.61
After	91.42 ± 11.69	89.08 ± 9.18*	91.10 ± 11.13	92.22 ± 12.43
Hip circumference				
Before	104.58 ± 7.16	102.68 ± 6.31	103.24 ± 6.83	104.74 ± 9.26
After	103.42 ± 7.36	102.54 ± 6.19	102.72 ± 6.80	104.54 ± 9.78
Waist to hip ratio				
Before	0.89 ± 0.08	0.90 ± 0.06	0.89 ± 0.08	0.89 ± 0.06
After	0.88 ± 0.08	0.87 ± 0.06*	0.89 ± 0.07	0.88 ± 0.06
HDL cholesterol				
Before	51.24 ± 15.46	50.90 ± 15.45	48.94 ± 13.91	49.10 ± 13.47
After	45.95 ± 9.79*	56.25 ± 14.11*	54.09 ± 12.36	56.00 ± 15.63
LDL cholesterol				
Before	132.57 ± 29.78	121.69 ± 25.01	120.50 ± 29.35	118.71 ± 28.01
After	124.18 ± 30.80	115.33 ± 24.56	118.14 ± 28.16	122.17 ± 25.76
Systolic pressure				
Before	120.64 ± 16.87	116.23 ± 13.88	123.57 ± 18.81	119.64 ± 15.02
After	117.25 ± 18.03	114.75 ± 14.03	124.14 ± 22.00	115.77 ± 14.75
Diastolic pressure				
Before	77.71 ± 13.19	77.08 ± 8.53	78.57 ± 16.18	76.29 ± 10.87
After	78.08 ± 14.96	75.42 ± 9.42	82.36 ± 17.23	72.85 ± 11.16
Triglycerides				
Before	137.71 ± 88.88	101.17 ± 78.86	103.64 ± 37.36	95.36 ± 54.21
After	111.73 ± 49.70	96.00 ± 42.33	142.50 ± 107.73	91.17 ± 45.00
Fasting glucose (mg/dL)				
Before	95.21 ± 45.62	83.00 ± 6.86	97.29 ± 37.11	87.43 ± 6.76
After	95.18 ± 52.59	78.58 ± 4.60*	92.71 ± 42.17	87.00 ± 9.90
HbA1c				
Before	5.79 ± 1.46	5.33 ± 0.28	5.77 ± 1.58	5.56 ± 0.43
After	6.05 ± 2.15	5.33 ± 0.30	5.82 ± 1.54	5.56 ± 0.43
BMI				
Before	26.86 ± 4.58	26.00 ± 3.75	26.87 ± 4.81	26.85 ± 5.35
After	26.62 ± 4.66	25.69 ± 3.68	26.60 ± 5.02	26.53 ± 5.29

Data presented as mean ± SD. * Significant difference compared with baseline ($p < 0.05$).

Supplementary Table S2. Mood disturbance before and after the intervention by group.

	Placebo (n =14)	Dietary fiber (n =13)	Probiotic (n =14)	Combined (n = 14)
Total mood disturbance (TMD)				
Before	105.57 ± 11.09	100.08 ± 20.30	98.36 ± 16.00	103.93 ± 21.96
After	108.00 ± 21.63	86.77 ± 11.81**	96.00 ± 18.43	90.36 ± 14.62*
Confusion				
Before	6.07 ± 4.78	4.62 ± 6.80	4.50 ± 5.45	5.64 ± 6.58
After	9.36 ± 6.15*	1.15 ± 1.41	4.14 ± 6.20	3.00 ± 3.40
Fatigue				
Before	8.36 ± 4.75	7.62 ± 6.63	7.43 ± 5.98	8.29 ± 5.37
After	8.86 ± 4.99	3.77 ± 2.55*	5.71 ± 4.34	5.71 ± 5.00

Anger				
Before	3.79 ± 3.96	4.15 ± 5.89	3.79 ± 3.31	5.43 ± 6.10
After	6.50 ± 5.54	1.77 ± 1.96	3.79 ± 4.02	3.00 ± 3.46
Tension				
Before	3.57 ± 2.65	2.00 ± 1.73	2.86 ± 3.46	3.07 ± 3.38
After	4.36 ± 3.25	1.15 ± 1.34	2.93 ± 3.15	1.50 ± 1.65
Depression				
Before	1.71 ± 1.86	0.77 ± 1.17	0.64 ± 1.08	2.07 ± 2.76
After	2.57 ± 2.31	0.46 ± 0.88	1.36 ± 2.10	0.71 ± 1.33
Vigor				
Before	10.36 ± 3.10	11.31 ± 7.18	12.36 ± 5.05	12.14 ± 5.50
After	14.21 ± 3.45**	12.62 ± 5.74	13.43 ± 3.03	13.79 ± 5.35
Esteem				
Before	7.57 ± 2.71	7.77 ± 3.54	8.50 ± 2.50	8.43 ± 4.15
After	9.43 ± 2.74*	8.92 ± 4.57	8.50 ± 1.51	9.79 ± 3.91*

Data presented as mean ± SD. * Significant difference compared with baseline ($p < 0.05$). ** Significant difference compared with baseline ($p < 0.01$).

Supplementary Table S3. Sleep quality disturbance before and after the intervention by group.

	Placebo (n =14)	Dietary fiber (n =13)	Probiotic (n =14)	Combined (n = 14)
Global PSQI score				
Before	6.71 ± 2.46	4.92 ± 2.29	5.07 ± 2.81	5.21 ± 3.29
After	6.21 ± 2.49	3.62 ± 2.50**	5.21 ± 2.81	5.71 ± 3.73
Subjective sleep quality				
Before	1.79 ± 0.43	1.38 ± 0.51	1.43 ± 0.76	1.21 ± 0.80
After	1.50 ± 0.52	0.85 ± 0.69*	1.36 ± 0.63	1.21 ± 0.80
Sleep latency				
Before	1.14 ± 0.95	0.69 ± 0.85	0.50 ± 0.85	0.79 ± 0.89
After	1.07 ± 0.83	0.69 ± 0.63	0.71 ± 0.83	0.79 ± 0.89
Sleep duration				
Before	0.79 ± 0.89	0.92 ± 0.76	0.64 ± 0.74	0.79 ± 0.89
After	0.71 ± 0.73	0.54 ± 0.66*	0.71 ± 0.91	0.86 ± 0.86
Habitual sleep efficiency				
Before	0.50 ± 0.76	0.31 ± 0.48	0.29 ± 0.61	0.36 ± 0.84
After	0.43 ± 0.65	0.23 ± 0.44	0.43 ± 0.94	0.71 ± 1.07
Sleep disturbance				
Before	1.21 ± 0.43	1.00 ± 0.41	1.21 ± 0.70	1.07 ± 0.27
After	1.14 ± 0.36	1.00 ± 0.41	1.07 ± 0.47	1.21 ± 0.43
Use of sleeping medication				
Before	0.29 ± 0.83	0.00 ± 0.00	0.21 ± 0.80	0.07 ± 0.27
After	0.36 ± 0.93	0.00 ± 0.00	0.21 ± 0.80	0.21 ± 0.80
Daytime dysfunction				
Before	1.00 ± 0.68	0.62 ± 0.51	0.79 ± 0.80	0.93 ± 0.83
After	1.00 ± 0.55	0.31 ± 0.48	0.71 ± 0.91	0.71 ± 0.83

Data presented as mean ± SD. * Significant difference compared with baseline ($p < 0.05$). ** Significant difference compared with baseline ($p < 0.01$).
